# Global gene expression analysis of the mouse colonic mucosa treated with azoxymethane and dextran sodium sulfate

**DOI:** 10.1186/1471-2407-7-84

**Published:** 2007-05-17

**Authors:** Rikako Suzuki, Shingo Miyamoto, Yumiko Yasui, Shigeyuki Sugie, Takuji Tanaka

**Affiliations:** 1 Department of Oncologic Pathology, Kanazawa Medical University, 1-1 Daigaku, Uchinada, Ishikawa 920-0293, Japan; 2 Division of Food Science and Biotechnology, Graduate School of Agriculture, Kyoto University, Kyoto 606-8502, Japan

## Abstract

**Background:**

Chronic inflammation is well known to be a risk factor for colon cancer. Previously we established a novel mouse model of inflammation-related colon carcinogenesis, which is useful to examine the involvement of inflammation in colon carcinogenesis. To shed light on the alterations in global gene expression in the background of inflammation-related colon cancer and gain further insights into the molecular mechanisms underlying inflammation-related colon carcinogenesis, we conducted a comprehensive DNA microarray analysis using our model.

**Methods:**

Male ICR mice were given a single ip injection of azoxymethane (AOM, 10 mg/kg body weight), followed by the addition of 2% (w/v) dextran sodium sulfate (DSS) to their drinking water for 7 days, starting 1 week after the AOM injection. We performed DNA microarray analysis (Affymetrix GeneChip) on non-tumorous mucosa obtained from mice that received AOM/DSS, AOM alone, and DSS alone, and untreated mice at wks 5 and 10.

**Results:**

Markedly up-regulated genes in the colonic mucosa given AOM/DSS at wk 5 or 10 included Wnt inhibitory factor 1 (*Wif1*, 48.5-fold increase at wk 5 and 5.7-fold increase at wk 10) and plasminogen activator, tissue (*Plat*, 48.5-fold increase at wk 5), myelocytomatosis oncogene (*Myc*, 3.0-fold increase at wk 5), and phospholipase A2, group IIA (platelets, synovial fluid) (*Plscr2*, 8.0-fold increase at wk 10). The notable down-regulated genes in the colonic mucosa of mice treated with AOM/DSS were the peroxisome proliferator activated receptor binding protein (*Pparbp*, 0.06-fold decrease at wk 10) and the transforming growth factor, beta 3 (*Tgfb3*, 0.14-fold decrease at wk 10). The inflammation-related gene, peroxisome proliferator activated receptor γ (*Pparγ *0.38-fold decrease at wk 5), was also down-regulated in the colonic mucosa of mice that received AOM/DSS.

**Conclusion:**

This is the first report describing global gene expression analysis of an AOM/DSS-induced mouse colon carcinogenesis model, and our findings provide new insights into the mechanisms of inflammation-related colon carcinogenesis and the establishment of novel therapies and preventative strategies against carcinogenesis.

## Background

The development and progression of colon carcinogenesis in both humans and rodents are known to be caused by the accumulation of cancer-related gene alterations, which results in their altered expression. Such genes include oncogenes, tumor suppressor genes, and mismatch repair genes [1,2]. These changes could affect the expression of a variety of downstream genes such as those involved in the cell cycle, apoptosis, adhesion, and angiogenesis [3]. Although both sporadic colorectal cancer (CRC) and colitis-associated CRC share several molecular alterations, the frequency and timing of certain key molecular changes are different [4]. As for the chemically-induced colon carcinogenesis in rodents, the *β-catenin* gene is frequently mutated in adenocarcinomas induced by colonic carcinogens, azoxymethane (AOM) and 2-amino-1-methyl-6-phenylimidazo [4,5-*b*]-pyridine (PhIP) in rodents [2]. The immunohistochemical expression of inducible nitric oxide synthase (iNOS), cyclooxygenase (COX)-2, and β-catenin is markedly elevated in the AOM-induced CRC in rats [2].

In our recent series of studies on inflammation-related mouse colon carcinogenesis, where mice received a single dose of different colonic carcinogens, i.e., AOM, PhIP, and 1,2-dimethylhydrazine, followed by one week of exposure to 2% dextran sodium sulfate (DSS) in drinking water, numerous CRCs developed within 20 weeks [5-7]. We also observed different sensitivities to AOM/DSS-induced colon carcinogenesis among 4 different strains (Balb/c, C3H/HeN, C57BL/6N, and DBA/2N) of mice [8]. Furthermore, numerous colonic tumors developed within 5 weeks in male and female *Apc*^*Min*/+ ^mice, which contain a truncating mutation in the *Apc *gene, when they received DSS in drinking water for 7 days [9]. Molecular analysis revealed a high-incidence (79–100%) of β-*catenin *gene mutations in induced colonic adenocarcinomas [6,7]. However, there were no mutations in the colonic adenocarcinomas developed in the *Apc*^*Min*/+ ^mice receiving DSS [9]. We therefore hypothesize that a powerful tumor-promotion effect of DSS is due to DSS-induced inflammatory stimuli, especially iNOS expression, since the incidence and multiplicity of these CRCs correlated with the increased inflammation score and elevated iNOS expression [5-7].

Microarray technology is a powerful tool to determine simultaneously the expression profile of numerous genes and is rapidly becoming a standard technique, which can be used in research laboratories across the world [10,11]. The introduction of microarray techniques has dramatic implications on cancer research, since it allows analysis of the expression of multiple genes in concert and helps find reliable clinical parameters for cancer occurrence. Several recent large-scale studies of gene expression using microarrays could thus provide useful information of inflammatory bowel disease (IBD) and colon carcinogenesis [12-14].

In the current study, we conducted global gene expression analysis of the non-neoplastic (inflamed) colonic mucosa of mice treated with AOM/DSS, AOM alone or DSS alone, and untreated mice utilizing Affymetrix GeneChip analysis in order to identify the molecular events in the background of AOM/DSS-induced mouse colon carcinogenesis. Using our model [5], gene expression analysis was done at wks 5 and 10 of the experimental period, i.e. when a few precursor lesions for colonic adenocarcinoma develop [15].

## Methods

### Animals, chemicals and diets

Male Crj: CD-1 (ICR) mice (Charles River Japan, Inc., Tokyo) aged 5 weeks were used. A colonic carcinogen, AOM, was purchased from the Sigma-Aldrich Co. (St. Louis, MO, USA). DSS with a molecular weight of 36,000–50,000 was purchased from MP Biochemicals, LLC (Cat. no. 160110, Aurora, OH, USA). DSS for the induction of colitis was dissolved in distilled water at 2% (w/v). Pelleted CRF-1 (Oriental Yeast Co., Ltd., Tokyo, Japan) was used as the basal diet throughout the study.

### Experimental procedure

**Figure 1:**
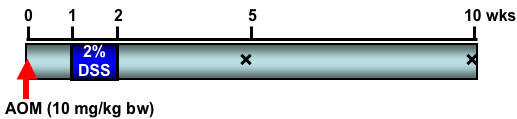
Experimental protocol for the AOM/DSS group.

A total of 40 mice were acclimated for 7 days with tap water and basal diet, CRF-1, *ad libitum*. The mice were divided into 4 groups, i.e., the AOM/DSS (10 mice, Figure 1), AOM alone (10 mice), DSS alone (10 mice), and untreated controls (10 mice). Five mice were analyzed at each time-point. In the AOM/DSS group, mice received a single i.p. injection of AOM (10 mg/kg body weight). Starting 1 week after the AOM injection, they were given 2% DSS in the drinking water for 7 days, without any further treatment until the end of the experiment. The AOM alone group was given a single i.p. injection of AOM (10 mg/kg body weight), and no further treatment. The DSS alone group was given 2% DSS in drinking water for 7 days (from wk 1 to wk 2), and was then maintained on basal diet and tap water. The untreated control group was maintained on basal diet and tap water throughout the experiment. All mice were maintained under the controlled conditions of humidity (50 ± 10%), light (12/12 h light/dark cycle), and temperature (23 ± 2°C) at Kanazawa Medical University Animal Facility according to the Institutional Animal Care Guidelines, and were killed by ether overdose at wks 5 and 10.

### Histopathological analysis

At autopsy, the large bowel was cut open longitudinally along the main axis, and washed with saline. After careful macroscopic inspection, the distal colon (0.5 cm from the anus) was cut and processed for histopathological examination after hematoxylin and eosin-staining. The remaining pieces of the colonic mucosa free from tumors and large ulcers was scraped for the microarray expression analysis.

### GeneChip analysis

Scraped colonic mucosa from each treated or untreated mice sacrificed at wks 5 and 10 were used for the GeneChip analysis. Microarray expression analysis was performed using a high-density oligonucleotide array (Affymetrix GeneChip array, Affymetrix, Santa Clara, CA, USA), and microarray expression analysis was done according to the instruction manual. In this study, 5 GeneChip array sets, corresponding to each mouse, were used for the individual time-points. The extraction of total mRNA from frozen colonic mucosa was done using TRIzol (Invitrogen Corporation, CA, USA). mRNA obtained from 5 mice at each time-point was hybridized to each array. Subsequently, the quality of RNA was evaluated using an Agilent 2100 Bioanalyzer (Agilent Technologies, Inc., Palo Alto, CA, USA), each RNA sample was converted into double-stranded cDNA by M-MLV ReverseTranscriptase (RNase H free) (TAKARA BIO Inc., Shiga, Japan) using GeneChip T7-Oligo(dT) Promoter Primer Kit. (Affymetrix, Santa Clara, CA, USA). Double-stranded cDNA was converted into double-stranded cRNA and biotinylated using GeneChip Expression 3'-Amplification Reagents for IVT labeling kit (Affymetrix). Biotin-labeled cRNA was purified by the GeneChip Sample Cleanup Module kit (Affymetrix) and fragmented. Hybridization of biotin-labeled cRNA fragment to Mouse Genome 430 2.0 array, washing, staining with streptavidin-phycoerythrin (Molecular Probes), and signal-amplification were performed according to the manufacturer's instructions. Mouse Genome 430 2.0 array has 45,000 probe sets for analyzing the expression of 39,000 transcripts and variants from over 34,000 well characterized mouse genes. Each hybridized Affymetrix GeneChip^® ^array was scanned with a GeneChip Scanner 3,000 and analyzed with the GeneChip Operating Software package version 1.2 (Affymetrix). We analyzed 40 array data sets (n = 5 for each time-point) to search for genes whose expression levels were altered among the groups. The average hybridization intensity for each array was determined using the Tukey's Biweight Estimate method. Prior to statistical analysis, 45,102 microarray data in each sample were identified both "Present" for Detection Call or "Increase" for Change Call based on a software analysis. The signal intensity of all array data sets at each time-point was compared among the groups, and the genes, which were differently expressed between the treatment and untreated groups at > 2-fold or < 1/2-fold, were selected for Venn diagrams. In addition, we used the significance analysis of microarrays (SAM) [16] method to identify differentially expressed genes in the treated and untreated groups. Significant regulation was defined as a fold change between the treatment and untreated control groups greater than 3 or less than 1/3. SAM that uses modified *t *test statistics for each gene of a dataset is a statistical technique for the finding of significant genes in a set of microarray experiments. SAM uses repeated permutations of the data to determine if the expression of any gene is significantly related to the response variable. A small fudge factor is added to the denominator in calculating the *t *value, thereby controlling for unrealistically low standard deviations in the tested gene. Furthermore, SAM allows control of the false discovery rate (FDR) [17] by setting a threshold to the difference between the actual test result and the result from repeated permutations of the tested groups. Thus, for genes called significant, SAM can estimate the percentage of genes identified by chance, FDR.

## Results

### Histopathology of the distal colon

**Figure 2:**
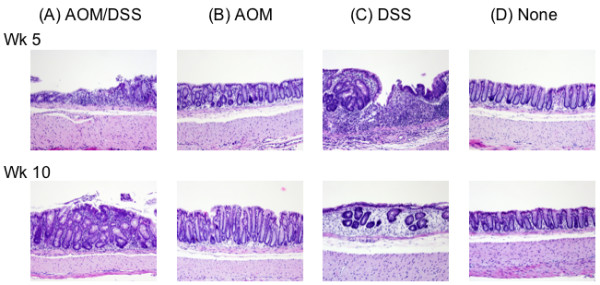
**Histopathology of colonic mucosa**. Colon from mice that received (A) AOM and 2%

Histopathological examination on the colonic mucosa revealed a few of spotted mucosal ulcers with regenerative changes in the AOM/DSS and DSS groups, but not in the AOM alone and untreated groups (Figure 2).

### Gene expression profile

**Figure 3:**
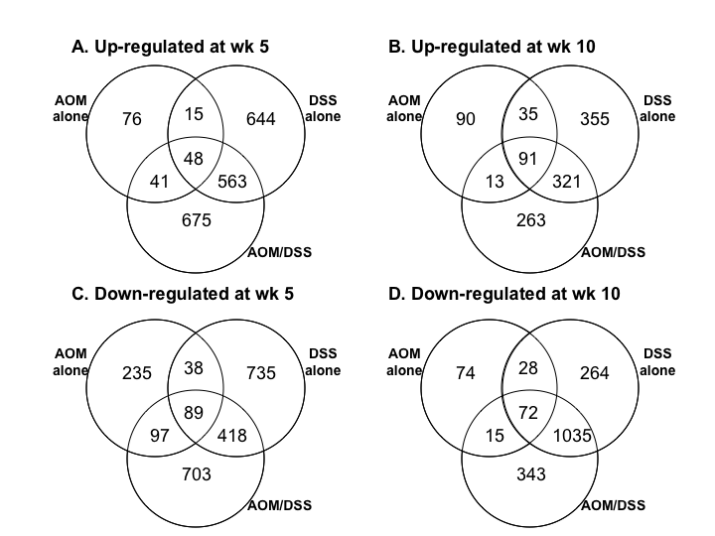
**Venn diagrams**. The numbers of up-regulated genes at wk 5 (A) and wk 10 (B), and down-regulated genes at wk 5 (C) and at wk 10 (D) in the colonic mucosa of mice in the AOM alone, DSS alone, and AOM/DSS groups. The numbers indicate the numbers of genes with their expression by over 2-fold up-regulated or by less 1/2-fold down-regulated between the treated (AOM-, DSS- or AOM/DSS) groups and untreated control group.

The numbers of up- (> 2-fold) or down- (< 1/2-fold) regulated genes in the colonic mucosa of mice were noted at the 2 time-points, as shown in the Venn diagrams (Figure 3). At wk 5, the number of up (180 genes)- or down (459 genes)-regulated genes in the AOM alone group was smaller than that of the DSS alone (1270 up- and 1280 down-regulated genes) and the AOM/DSS treated groups (1327 up- and 1307 down-regulated genes). Similarly, at wk 10 genes with altered expression in the AOM alone group (229 up- and 189 down-regulated genes) was much less than in comparison the 2 other groups (688 up- and 1465 down-regulated genes in the AOM/DSS group; and, 802 up- and 1399 down-regulated genes in the DSS alone group). The numbers of over- or under-expressed genes (611 up- and 507 down-regulated genes at wk 5; and 412 up- and 1107 down-regulated genes at wk 10), which were found in both the DSS alone and AOM/DSS groups at the 2 two time-points, were greater than that detected in both the AOM alone and AOM/DSS-treated groups (89 up- and 186 down-regulated genes at wk 5; and 104 up- and 87 down-regulated genes at wk 10). Regarding the AOM/DSS group, the number of genes (675 up-regulated; and 703 down-regulated) with altered expression at wk 5 was greater than that found at wk 10 (263 up-regulated; and 343 down-regulated).

### Up-regulated genes in the AOM/DSS group

Among the > 2-fold up-regulated genes in the AOM/DSS group (Figure 3A and 3B), 163 and 35 genes were significantly elevated by > 3-fold at wks 5 and 10, respectively. Among them, the genes whose functions are known are listed in Tables 1 and 2. The expression of Wnt inhibitory factor 1 (*Wif1*) and plasminogen activator, tissue (*Plat*) was up-regulated by 48.5-fold at wk 5 (Table 1). In addition, myelocytomatosis oncogene (*Myc*), matrix metalloproteinase 2 (*Mmp2*) and 14 (*Mmp14*) were significantly up-regulated by 3.0~ 4.0-fold at wk 5. However, at wk 10, phospholipase A2, group IIA (platelets, synovial fluid) (*Plscr2*), which plays a key role in the production of pro-inflammatory mediators, was significantly up-regulated by 8.0-fold (Table 2). *Axin 2*, T-box 3 (*Tbx3*), chloride channel calcium activated 1 /// Chloride channel calcium activated 2 (*Clca1 */// *Clca2*), and ***W**if1* were also significantly up-regulated by 3.0~ 48.5-fold at wks 5 and 10 (Tables 1 and 2).

### Down-regulated gense in the AOM/DSS group

Among the down-regulated genes by < 1/2-fold in the AOM/DSS group (Figure 3C), 114 genes were significantly down-regulated by < 1/3-fold at wk 5. The genes with known functions are listed in Table 3. Genes associated with the transport, regulation of transcription, and proteolysis and peptidolysis were suppressed. At wk 10, among the genes that were < 1/2-fold (Figure 3D), 6 genes with known functions were significantly down-regulated by < 1/3-fold in the AOM/DSS group (Table 4). The gene with markedly less expression was peroxisome proliferator activated receptor binding protein (*Pparbp*), which is co-activator of peroxisome proliferator activated receptor (*Ppar*).

### Expression of inflammation-related genes

The expression of inflammation-related genes was evaluated utilizing the Chip data (data not shown). At wk 5 or 10, tumor necrosis factor receptor superfamily, member 1b (*Tnfrsf1b*), interferon gamma inducible protein 47 (*Ifi47*), tumor necrosis factor, alpha-induced protein 9 (*Tnfaip9*), interferon gamma induced GTPase (*Igtp*), chemokine (C-C motif) receptor 1 (*Ccr1*), prostaglandin D2 synthase 2, hematopoietic (*Ptgds2*), transforming growth factor, beta 1 (*Tgfb1*), and toll-like receptor 2 (*Tlr2*) were up-regulated by > 2-fold in the AOM/DSS group compared to the untreated controls. In addition, transforming growth factor, beta 3 (*Tgfb3*) and peroxisome proliferator activated receptor γ (Pparγ) in the AOM/DSS group were down-regulated by < 1/2-fold when compared with the untreated group at wk 5 or 10.

### Up-regulated genes in colonic mucosa of all groups

Forty-eight and 91 genes were up-regulated ≥ 2-fold in common in the AOM alone, DSS alone, and AOM/DSS groups at wks 5 and 10, respectively (Figure 3A and 3B). At wk 5, the expression of runt related transcription factor 2 (*Runx2*) and secreted frizzled-related sequence protein 2 (*Sfrp2*) were significantly up-regulated by more than 10-fold in the AOM/DSS group. In addition, pancreatic lipase-related protein 2 (*Pnliprp2*) was significantly over-expressed in both the DSS alone and AOM/DSS groups. At wk 10, the expression of suppressor of cytokine signaling 3 (*Socs3*) and interferon gamma inducible protein 47 (*Ifi47*) were significantly up-regulated by nearly 10-fold in the AOM/DSS group. The genes responsible for nitrogen metabolism and nitric oxide biosynthesis were also significantly up-regulated in the DSS alone and AOM/DSS group at wk 10. The up-regulation of interferon-induced protein with tetratricopeptide repeats 2 (*Ifit2*) was found at wks 5 and 10.

### Down-regulated gene in colonic mucosa of AOM/DSS, AOM alone, and DSS alone groups

The genes down-regulated by < 1/2-fold in common in the AOM alone, DSS alone, and AOM/DSS groups at wk 5 and 10 numbered 89 and 72, respectively (Figure 3C and 3D). The genes associated with the transport and regulation of transcription were down-regulated in all the treatment groups at wks 5 and 10. In the AOM/DSS group, tensin-like SH2 domain containing 1 (*Tens1*), ring finger protein 25 (*Rnf25*), carbonic anhydrase 8 (*Car8*), solute carrier family 13 (sodium/sulphate symporters), member 1 (*Slc13a1*), regulator of G-protein signaling 17 (*Rgs17*), prolactin receptor (*Prlr*), and complement receptor related protein (*Crry*) were significantly down-regulated at wk 5. At wk 10, xanthine dehydrogenase (*Xdh*), Max dimerization protein (*Mad*), protein kinase, cAMP dependent, catalytic, beta (*Prkacb*), plexin A2 (*Plxna2*), and 4.5 LIM domains 1 (*Fhl1*) in the DSS alone and AOM/DSS groups significantly suppressed their expression in comparison with the AOM alone group. At both wks 5 and 10, kit ligand (*Kitl*) showed significantly reduced expression.

## Discussion

The current investigation using GeneChip analysis demonstrates the alterations in multiple genes' expression in the colonic mucosa of mice treated with AOM and/or DSS. Although we found an over-expression of *β-catenin*, COX-2 and iNOS in the colonic neoplasms induced by the treatment of AOM and DSS in mice in our previous immunohistochemical studies [5-7], other numerous and attractive gene alterations became apparent in the present study. Interestingly, the number of genes that showed altered expression in the colonic mucosa in mice exposed to AOM/DSS was greater than that found in the mice given AOM alone or DSS alone. We also revealed that the number of genes with altered expression in the colonic mucosa in the mice treated with AOM/DSS at wk 5 was greater than that detected at wk 10. Our findings may suggest the numerous gene alterations at the early phase of colitis-related mouse colon carcinogenesis might contribute to the development of colonic tumors at the later stage of carcinogenesis.

The most striking change in gene expression observed was over-expression of *Wif1 *(48.5-fold increase over the untreated control) seen in the AOM/DSS group at wk 5. The Wingless-type (Wnt) signaling pathway is known to play a central role in CRC development [18]. Wif1 is a secreted antagonist that can bind to Wnt proteins directly, and thus inhibits the Wnt signaling pathway [19]. Down-regulation of ***W**if1* mRNA expression is observed in esophageal, gastric, colorectal, and pancreatic cancers [20]. These findings in cancer tissue specimens conflict with our results, but our findings were from colonic mucosa without tumors. We can thus speculate that the over-expression of ***W**if1 *prevents the carcinogenesis process through inactivation of Wnt signaling, since the up-regulation of Wif1 was found in the AOM/DSS group, but not in the AOM alone and DSS alone groups. On the other hand, Cebrat et al. [21] suggested that ***W**if1* may potentially be a new factor in intestinal tumorigenesis. They reported that *Wif1 *is over-expressed in intestinal adenomas of *Apc*^*Min*/+ ^mice and human colon adenocarcinoma cell lines [21]. In the present study, *Wif1 *is over-expressed at wks 5 and 10. The up-regulation of *Wif1 *is thus one of the important genes in this AOM/DSS-induced mouse colon carcinogenesis. Further investigations are required to clarify how *Wif1 *is involved in the inflammation-related colon carcinogenesis. *Plat *was also up-regulated in the AOM/DSS group. Although we did not determine the degree of inflammation, the findings are in agreement with those reported by others [22] and with our own data [23].

One of the interesting findings is that the stress-related genes, i.e., metallothionein (*Mt*) and heat shock protein (*HSP*) were up-regulated. Over-expression of *Mt* and *HSP *was in the inflamed colonic mucosa of mice and rats that received DSS [24,25]. The *Mt *expression is also altered in the early step of IBD and ulcerative colitis (UC)-associated CRC [26]. Costello et al. [12] reported over-expression of *HSP* in the colon in Crohn's disease (CD), which is another type of IBD. Since the oxidative/nitrosative stress caused by DSS in the colonic mucosa contributes to colonic tumor development in our inflammation-related mouse colon carcinogenesis model [8,9,15,23], *Mt-3 *might be up-regulated to protect cells from oxidative stress [27].

In the present study, genes involving inflammation altered their expression in the AOM/DSS group, for example, prostaglandin-endoperoxide synthase 2 (*Ptgds2*), which is one of the important mediators of colonic inflammation [28], was up-regulated. However, the expression of *Ptgs2* did not significantly alter in the AOM/DSS group when compared with the untreated group between wks 5 (1.6-fold increase) and 10 (0.5-fold increase). As for nitric oxide synthases, the expression of nitric oxide synthase 2, inducible macrophage (*NOS2*), but not *NOS1 *and *NOS3*, was up-regulated by 3.6-fold at wk 5. Moreover, this up-regulation continued up to wk 10 (by 1.6-fold), being in line with our previous findings [15]. Also, pancreatic lipase-related protein (*pnliprp2*) and *Plscr2 *were over-expressed in this study. Dietary triglycerides are precursors for cellular membranes and for prostaglandins, thromboxanes, and leukotrienes [29]. Triacylglycerols and phospholipids are hydrolyzed by pancreatic enzymes, including pancreatic lipase and phospholipase A_2 _(*PLA*_2_). *PLA*_2_is a rate-limiting enzyme of the arachidonic acid cascade, and is involved in the production of prostaglandins. In addition, *PLA*_2_influences intestinal inflammation in human [13] and rodents [30]. *PLA*_2_also involves colitis-related colon carcinogenesis in rats [31]. These genes might thus play a pivotal role in the inflammatory processes in our mouse model used in this study. PPARγ plays certain role in anti-inflammation [32], colon carcinogenesis [33,34], and cancer development in the inflamed colon [33,35]. In fact, lowered expression of PPARγ could be a risk factor for carcinogenesis [36,37]. Therefore, down-regulated PPARγ by the combined treatment of AOM and DSS in this study might partly contribute to CRC development [5,8,15,23]. In the current study, one of the striking down-regulated genes was *Pparbp*, which is identified as a coactivator for PPARγ [38]. Down-regulated *Pparbp* might be partly associated with down expression of PPARγ. Loss of transforming growth factor β (TGF-β) signaling is considered to be an essential step in carcinogenesis [39], and decreased TGF-β_3 _mRNA level is mediated by nitric oxide [40]. Expression of cytochrome P450 (CYP) is altered during inflammation [41]. Administration of lipopolysaccharide that causes inflammation reduces the intestinal epithelial CYP3A [42] and hepatic CYP2C [43] activities in rats. In the current study, CYP 3A and 2C family (CYP, family 3, subfamily a, polypeptide 13 (*Cyp3a13*) and CYP, family 2, subfamily c, polypeptide 55 (*Cyp2c55*)) were down-regulated by the combined treatment with AOM and DSS at wks 5 and 10. Down-regulation of CYP, family 4, subfamily f, polypeptide 16 (*Cyp4f16*) genes could induce inflammatory cytokines and mediators in the colon of mice treated with AOM/DSS, since the concentrations of leukotriene and prostaglandin mediators are elevated by decreased CYP 4F level [44]. Alteration of inflammatory mediators produced by treatment with AOM/DSS may enhance or accelerate the occurrence and progression of CRC.

The histopathological relationship between the severity of inflammation induced by DSS and CRC development in mice is closely similar to that of UC and CRC occurrence in humans [45,46]. Alterations of gene expression in the colonic mucosa of mice treated with AOM/DSS were similar to those found in the IBD. Over-expression of *PAP*, *Mmp 2*, *Mmp 14*, *Myc*, *MT*, and *Plscr2 *was found in the colonic mucosa of IBD patients [12,13,47-49]. EGL nine homolog 3 (*C. elegans*) (*Egln3*) and *Plat* gene were also up-regulated in the colon of UC patients [12,22]. Down-regulation of meprin 1 alpha (*Mep 1a*) and solute carrier family 20, member 1 (*Slc20a1*) were observed in the colon of UC [13]. Furthermore, alterations of gene expression in interleukin 1 receptor antagonist 1 (*Il1rn*, 2-fold), enoyl coenzyme A hydratase 1, peroxisomal (*Ech1*, 0.47-fold), immediate early response 3 (*Ler3*, 3.8-fold), baculoviral IAP repeat-containing 4 (*Birc4*, 0.41-fold), DnaJ (*Hsp40*) homolog, subfamily B, member 5 (*Dnajb5*, 2.6-fold), neural precursor cell expressed, developmentally down-regulated gene 9 (*Nedd9*, 0.44-fold), cytokine inducible SH2-containing protein (*Cish*, 0.5-fold), centromere protein E (*Cenpe*, 2.1-fold), and tissue inhibitor of metalloproteinase 2 (*Timp2*, 2.1-fold) in the AOM/DSS group were similar to those found in the UC and/or CD patients [12,13,50]. These findings may suggest that our model is useful for a mechanistic analysis and therapeutic approaches of IBD-related CRC.

Nuclear factor-kappaB (NF-κB) is a transcription factor that plays a crucial role in inflammation, immunity, cell proliferation, apoptosis, and tumorigenesis [51]. Activation of NF-κB is associated with transglutaminase 2, *MT*, and tumor necrosis factor receptor [51-53], which was up-regulated in the present study. Oxidative stress involving in CRC development in the AOM/DSS-induced mouse-colon carcinogenesis [8,15,23] also leads to NF-κB activation [54]. Certain genes and their products that involve in tumorigenesis are regulated by NF-κB [51]. iNOS and COX-2 that are up-regulated in colonic neoplasms induced by AOM and DSS [5], might be influenced by the activation NF-κB [51]. It is thus possible that NF-κB also plays an important role in AOM/DSS-induced mouse colon carcinogenesis.

Our GeneChip analysis of the gene expression in the colonic mucosa of mice that received AOM/DSS first revealed altered expression of multiple genes. The gene expressional profile of the AOM/DSS group was dissimilar to that of the AOM alone or DSS alone group. These are the genes whose transcription was more affected by the stimulus of AOM/DSS treatment in comparison to those with AOM or DSS treatment alone. Our results shed further light on the mechanisms of inflammation-related colon carcinogenesis. The expression of several genes, including those classified as proteolysis and peptidolysis, cell adhesion, transport, regulation of cell growth, development, DNA replication, and regulation of transcription (DNA-dependent), were altered during the early phase (wks 5–10) of AOM/DSS-induced mouse colon tumorigenesis. Based on our results, further investigations are underway to identify and confirm the optimal target genes that involve in inflammation-related colon carcinogenesis in humans as well as rodents in our laboratory.

## Conclusion

Our findings by global genes' expression analysis for an AOM/DSS-induced mouse colon carcinogenesis model probably provide new insights into the mechanisms of inflammation-related colon carcinogenesis and the establishment of new therapies and preventative strategies for inflammation-related colon carcinogenesis.

## Abbreviations

AOM, azoxymethane; DSS, dextran sodium sulfate; CRC, colorectal cancer; PhIP, 2-amino-1-methyl-6-phenylimidazo [4,5-*b*]-pyridine; iNOS, inducible nitric oxide synthase; COX-2, cyclooxygenase; IBD, inflammatory bowel disease; PPAR, peroxisome proliferator activated receptor; Wnt, Wingless-type; UC, ulcerative colitis; CD, Crohn's disease; PLA_2_, phospholipase A_2_; TGF-β, transforming growth factor β; CYP, cytochrome P450; NF-κB, Nuclear factor-kappaB.

## Competing interests

The author(s) declare that they have no competing interests.

## Authors' contributions

RS co-designed the study with TT, wrote the manuscript, carried out the animal study, and data analysis. SM performed the animal study and data analysis. YY performed the animal study and data analysis. SS carried out data analysis. TT guided the study concept and design, revised final submission, and helped with data interpretation. All the authors read and approved the final manuscript.

## Pre-publication history

The pre-publication history for this paper can be accessed here:



## Figures and Tables

**Table 1:** The up-regulated genes by over 3-fold in the AOM/DSS group at wk 5

Gene name (gene symbol)	Average fold of change (AOM/DSS vs. untreated)
Plasminogen activator, tissue (*Plat*)	48.5
Wnt inhibitory factor 1 (*Wif1*)	48.5
Metallothionein 3 (*Mt3*)	11.3
Chloride channel calcium activated 1 /// chloride channel calcium activated 2 (*Clca1 */// *Clca2*)	9.2
Nuclear factor, erythroid derived 2, like 3 (*Nfe2l3*)	7.5
Protein tyrosine phosphatase, receptor type, O (*Ptpro*)	7.5
Transient receptor potential cation channel, subfamily V, member 6 (*Trpv6*)	7
Chloride channel calcium activated 2 (*Clca2*)	7
Dual specificity phosphatase 4 (*Dusp4*)	5.7
Heat shock protein 1 (*Hspb1*)	5.7
Advillin (*Avil*)	5.3
Proprotein convertase subtilisin/kexin type 9 (*Pcsk9*)	4.9
Extracellular matrix protein 1 (*Ecm1*)	4.9
Bone morphogenetic protein 4 (*Bmp4*)	4.6
Insulin-like growth factor binding protein 3 (*Igfbp3*)	4.3
Axin2 (*Axin2*)	4.3
Transferring (*Trf*)	4.3
Caldesmon 1 (*Cald1*)	4.3
Guanylate nucleotide binding protein 2 (*Gbp2*)	4.3
Gap junction membrane channel protein alpha 1 (*Gja1*)	4.3
WASP family 1 (*Wasf1*)	4
Aspartoacylase (aminoacylase) 2 (*Aspa*)	4
Notch gene homolog 4 (Drosophila) (*Notch4*)	4
Matrix metalloproteinase 2 (*Mmp2*)	4
Actin, alpha 2, smooth muscle, aorta (*Acta2*)	4
Transgelin (*Tagln*)	4
Dermatopontin (*Dpt*)	4
Integrin alpha X (*Itgax*)	4
Hedgehog-interacting protein (*Hhip*)	4
Latent transforming growth factor beta binding protein 4 (*Ltbp4*)	4
Myeloid ecotropic viral integration site-related gene 1 (*Mrg1*)	3.7
Dachshund 1 (Drosophila) (*Dach1*)	3.7
Membrane targeting (tandem) C2 domain containing 1 (*Mtac2d1*)	3.7
Laminin B1 subunit 1 (*Lamb1-1*)	3.7
RAS-related C3 botulinum substrate 3 (*Rac3*)	3.7
Nitric oxide synthase 2, inducible, macrophage (*Nos2*)	3.6
Bone morphogenetic protein 5 (*Bmp5*)	3.5
Methylthioadenosine phosphorylase (*Mtap*)	3.5
Transcription factor 4 (*Tcf4*)	3.5
Transcription factor 21 (*Tcf21*)	3.5
CCAAT/enhancer binding protein (C/EBP), delta (*Cebpd*)	3.5
Apolipoprotein B editing complex 2 (*Apobec2*)	3.5
Microtubule associated serine/threonine kinase-like (*Mastl*)	3.5
Carboxypeptidase X 2 (M14 family) (*Cpxm2*)	3.5
Ubiquitin specific protease 18 (*Usp18*)	3.5
Procollagen, type VI, alpha 2 (*Col6a2*)	3.5
Chloride channel calcium activated 4 (*Clca4*)	3.5
Myosin, light polypeptide 9, regulatory (*Myl9*)	3.5
Small muscle protein, X-linked (*Smpx*)	3.5
Glutathione peroxidase 3 (*Gpx3*)	3.5
Septin 4 (*4-Sep*)	3.5
Cysteine rich protein 2 (*Crip2*)	3.5
Fibroblast growth factor 7 (*Fgf7*)	3.2
Proline arginine-rich end leucine-rich repeat (*Prelp*)	3.2
Expressed sequence AW125753 /// protein tyrosine phosphatase, receptor type, J (*AW125753 */// *Ptprj*)	3.2
BTB (POZ) domain containing 11 (*Btbd11*)	3.2
Calpain 2 (*Capn2*)	3.2
Transglutaminase 2, C polypeptide (*Tgm2*)	3.2
Matrix metalloproteinase 14 (membrane-inserted) (*Mmp14*)	3.2
SEC8-like 1 (S. cerevisiae) (*Sec8l1*)	3.2
ADP-ribosylation factor 2 (*Arf2*)	3.2
Calponin 3, acidic (*Cnn3*)	3.2
Actin, gamma 2, smooth muscle, enteric (*Actg2*)	3.2
Stathmin 1 (*Stmn1*)	3.2
Chondroitin sulfate proteoglycan 2 (*Cspg2*)	3.2
DEP domain containing 1a (*Depdc1a*)	3.2
Inhibitor of DNA binding 2 (*Idb2*)	3.2
Pleckstrin homology-like domain, family A, member 1 (*Phlda1*)	3.2
Myelocytomatosis oncogene (*Myc*)	3
Stabilin 1 (*Stab1*)	3
Guanylate cyclase 1, soluble, beta 3 (*Gucy1b3*)	3
RNA binding motif, single stranded interacting protein 1 (*Rbms1*)	3
RAD51 associated protein 1 (*Rad51ap1*)	3
SRY-box containing gene 4 (*Sox4*)	3
T-box 3 (*Tbx3*)	3
Kruppel-like factor 7 (ubiquitous) (*Klf7*)	3
Bcl-2-related ovarian killer protein (*Bok*)	3
Estrogen-related receptor beta like 1 (*Esrrbl1*)	3
Complement component 3a receptor 1 (*C3ar1*)	3
Tropomyosin 2, beta (*Tpm2*)	3
Procollagen, type V, alpha 1 (*Col5a1*)	3
Integrin alpha 8 (*Itga8*)	3
Annexin A8 (*Anxa8*)	3
Podocalyxin-like (*Podxl*)	3
Retinol dehydrogenase 9 (*Rdh9*)	3
Tumor necrosis factor, alpha-induced protein 9 (*Tnfaip9*)	3

**Table 2:** The up-regulated genes by over 3-fold in the AOM/DSS group at wk 10

Gene name (gene symbol)	Average fold of change (AOM/DSS vs. untreated)
Pancreatic lipase-related protein 2 (*Pnliprp2*)	14.9
Matrix gamma-carboxyglutamate (gla) protein (*Mglap*)	9.8
Phospholipase A2, group IIA (platelets, synovial fluid) (*Plscr2*)	8
Wnt inhibitory factor 1 (*Wif1*)	5.7
Interferon induced transmembrane protein 3 (*Ifitm3*)	4.3
SRY-box containing gene 17 (*Sox17*)	4
Expressed sequence AW111922	4
T-box 3 (*Tbx3*)	3.7
Interferon gamma induced GTPase (*Igtp*)	3.7
EGL nine homolog 3 (C. elegans) (*Egln3*)	3.5
Roundabout homolog 1 (Drosophila) (*Robo1*)	3.5
Histocompatibility 2, Q region locus 1 (*H*2-*Q*1)	3.5
Glutamyl aminopeptidase (*Enpep*)	3.2
Chloride channel calcium activated 1 (*Clca1*)	3.2
Chloride channel calcium activated 1 /// chloride channel calcium activated 2 (*Clca1 */// *Clca2*)	3.2
Defensin related sequence cryptdin peptide (paneth cells) (*Defcr-rs1*)	3.2
Axin2 (*Axin2*)	3
Fatty acid desaturase 1 (*Fads1*)	3
ATPase, class V, type 10A (*Atp10a*)	3
CD8 antigen, alpha chain (*Cd8a*)	3
Tumor necrosis factor, alpha-induced protein 9 (*Tnfaip9*)	3

**Table 3:** The down-regulated genes by less than 1/3-fold in the AOM/DSS group at wk 5

Gene name (gene symbol)	Average fold of change (AOM/DSS vs. untreated)
Solute carrier family 10, member 2 (*Slc10a2*)	0.12
Rho GTPase activating protein 20 (*Arhgap20*)	0.12
Cytochrome P450, family 2, subfamily d, polypeptide 26 (*Cyp2d26*)	0.13
Aldehyde dehydrogenase family 1, subfamily A1 (*Aldh1a1*)	0.14
Dipeptidase 1 (renal) (*Dpep1*)	0.15
UDP-glucuronosyltransferase 8 (*Ugt8*)	0.15
Dehydrogenase/reductase (SDR family) member 8 (*Dhrs8*)	0.15
Solute carrier family 3, member 1 (*Slc3a1*)	0.16
Paired-like homeodomain transcription factor 2 (*Pitx2*)	0.19
Glycoprotein galactosyltransferase alpha 1, 3 (*Ggta1*)	0.19
N-acetylglutamate synthase (*Nags*)	0.19
Adrenergic receptor, alpha 2a (*Adra2a*)	0.19
3-hydroxy-3-methylglutaryl-Coenzyme A synthase 2 (*Hmgcs2*)	0.2
Cytochrome P450, family 4, subfamily f, polypeptide 16 (*Cyp4f16*)	0.2
Glucosaminyl (N-acetyl) transferase 3, mucin type (*Gcnt3*)	0.2
Glutamyl aminopeptidase (*Enpep*)	0.2
Homeo box B5 (*Hoxb5*)	0.22
Caudal type homeo box 2 (*Cdx2*)	0.22
Fatty acid binding protein 2, intestinal (*Fabp2*)	0.22
Glutamic pyruvic transaminase 1, soluble (*Gpt1*)	0.22
Mitogen activated protein kinase 10 (*Mapk10*)	0.23
Meprin 1 alpha (*Mep1a*)	0.23
UDP-N-acetyl-alpha-D-galactosamine:(N-acetylneuraminyl)-galactosylglucosylceramide-beta-1, 4-N-acetylgalactosaminyltransferase (*Galgt1*)	0.23
CD36 antigen (*Cd36*)	0.25
Mercaptopyruvate sulfurtransferase (*Mpst*)	0.25
FK506 binding protein 5 (*Fkbp5*)	0.27
Acetyl-Coenzyme A acyltransferase 2 (mitochondrial 3-oxoacyl-Coenzyme A thiolase) (*Acaa2*)	0.27
Histocompatibility 2, class II antigen E alpha (*H2-Ea*)	0.27
RAB37, member of RAS oncogene family (*Rab37*)	0.27
BRAF35/HDAC2 complex (*Bhc80*)	0.29
Protein tyrosine phosphatase, receptor type, R (*Ptprr*)	0.29
N-acylsphingosine amidohydrolase (alkaline ceramidase) 3 (*Asah3*)	0.29
Pyruvate dehydrogenase kinase, isoenzyme 4 (*Pdk4*)	0.31
Nuclear receptor subfamily 5, group A, member 2 (*Nr5a2*)	0.31
RIKEN cDNA 9030612M13 gene (*9030612M13Rik*)	0.31
Mitogen activated protein kinase kinase kinase 2 (*Map3k2*)	0.31
Angiotensin I converting enzyme (peptidyl-dipeptidase A) 2 (*Ace2*)	0.31
Vanin 1 (*Vnn1*)	0.31
ATP-binding cassette, sub-family B (MDR/TAP), member 1A (*Abcb1a*)	0.31
Kinesin family member C3 (*Kifc3*)	0.31
3'-phosphoadenosine 5'-phosphosulfate synthase 2 (*Papss2*)	0.33
Cytochrome P450, family 2, subfamily c, polypeptide 55 (*Cyp2c55*)	0.33
Ring finger protein 130 (*Rnf130*)	0.33
Phosphate cytidylyltransferase 1, choline, alpha isoform (*Pcyt1a*)	0.33
Potassium channel, subfamily K, member 5 (*Kcnk5*)	0.33
Solute carrier family 9 (sodium/hydrogen exchanger), member 2 (*Slc9a2*)	0.33
Solute carrier family 20, member 1 (*Slc20a1*)	0.33
Synaptotagmin-like 4 (*Sytl4*)	0.33
Regulator of G-protein signaling 13 (*Rgs13*)	0.33
Diaphanous homolog 1 (Drosophila) (*Diap1*)	0.33
Fibrinogen-like protein 2 (*Fgl2*)	0.33
Epidermal growth factor (*Egf*)	0.33

**Table 4:** The down-regulated genes by less than 1/3-fold in the AOM/DSS group at wk 10

Gene name (gene symbol)	Average fold of change (AOM/DSS vs. untreated)
RIKEN cDNA 1110033J19 gene	0.05
Peroxisome proliferator activated receptor binding protein (*Pparbp*)	0.06
Serine/threonine kinase 25 (yeast) (*Stk25*)	0.09
Transforming growth factor, beta3 (*Tgfb3*)	0.14
Cytochrome P450, family 3, subfamily a, polypeptide 13 (*Cyp3a13*)	0.31
APG4 (ATG4) autophagy-related homolog A (S. cerevisiae) (*Apg4a*)	0.33
Pleckstrin homology domain interacting protein (*Phip*)	0.33
